# *“Just too busy living in the moment and surviving”:* barriers to accessing health care for structurally vulnerable populations at end-of-life

**DOI:** 10.1186/s12904-019-0396-7

**Published:** 2019-01-26

**Authors:** K. I. Stajduhar, A. Mollison, M. Giesbrecht, R. McNeil, B. Pauly, S. Reimer-Kirkham, N. Dosani, B. Wallace, G. Showler, C. Meagher, K. Kvakic, D. Gleave, T. Teal, C. Rose, C. Showler, K. Rounds

**Affiliations:** 1 Institute on Aging and Lifelong Health, University of Victoria, 3800 Finnerty Road, Victoria, BC V8P 5C2 Canada; 2 BC Centre on Substance Use, 608–1081 Burrard Street, Vancouver, BC V6Z 1Y6 Canada; 3 Department of Medicine, University of British Columbia, 2775 Laurel Street, Vancouver, BC V5Z 1M9 Canada; 4 School of Nursing, University of Victoria, 3800 Finnerty Road, Victoria, BC V8P 5C2 Canada; 5 Canadian Institute for Substance Use Research, University of Victoria, 3800 Finnerty Road, Victoria, BC V8P 5C2 Canada; 6 School of Nursing, Trinity Western University, 7600 Glover Road, Langley, BC V2Y 1Y1 Canada; 7 Inner City Health Associates, 59 Adelaide St. E, Toronto, ON M5C 1K6 Canada; 8 School of Social Work, University of Victoria, 3800 Finnerty Road, Victoria, BC V8P 5C2 Canada; 9 Victoria Cool Aid Community Health Centre, 1st Floor, Access Health Centre, 713 Johnson Street, Victoria, BC V8W 1M8 Canada; 10 AIDS Vancouver Island, 713 Johnson St, Victoria, BC V8W 1M8 Canada

**Keywords:** Access to care, Structural vulnerability, Homelessness, EOL care, Health equity, Ethnographic methods, Canada

## Abstract

**Background:**

Despite access to quality care at the end-of-life (EOL) being considered a human right, it is not equitable, with many facing significant barriers. Most research examines access to EOL care for homogenous ‘normative’ populations, and as a result, the experiences of those with differing social positioning remain unheard. For example, populations experiencing structural vulnerability, who are situated along the lower rungs of social hierarchies of power (e.g., poor, homeless) will have unique EOL care needs and face unique barriers when accessing care. However, little research examines these barriers for people experiencing life-limiting illnesses *and* structural vulnerabilities. The purpose of this study was to identify barriers to accessing care among structurally vulnerable people at EOL.

**Methods:**

Ethnography informed by the critical theoretical perspectives of equity and social justice was employed. This research drew on 30 months of ethnographic data collection (i.e., observations, interviews) with structurally vulnerable people, their support persons, and service providers. Three hundred hours of observation were conducted in homes, shelters, transitional housing units, community-based service centres, on the street, and at health care appointments. The constant comparative method was used with data collection and analysis occurring concurrently.

**Results:**

Five significant barriers to accessing care at EOL were identified, namely: (1) The survival imperative; (2) The normalization of dying; (3) The problem of identification; (4) Professional risk and safety management; and (5) The cracks of a ‘silo-ed’ care system. Together, findings unveil inequities in accessing care at EOL and emphasize how those who do not fit the ‘normative’ palliative-patient population type, for whom palliative care programs and policies are currently built, face significant access barriers.

**Conclusions:**

Findings contribute a nuanced understanding of the needs of and barriers experienced by those who are both structurally vulnerable and facing a life-limiting illness. Such insights make visible gaps in service provision and provide information for service providers, and policy decision-makers alike, on ways to enhance the equitable provision of EOL care for *all* populations.

## Background

Access to quality care at end-of-life (EOL) is increasingly recognized as a human right [1], yet, many face significant barriers due to inequitable access [2–4]. Globally, it is estimated that only 14% of those in need receive palliative care [1], with barriers attributed to organizational factors and the deficiency of programs to deliver palliative care [2, 4]; the dearth of clear policies and educational programmes that teach palliative care [3]; a lack of public awareness about palliative care [4]; and family related issues, such as the absence of families’ willingness or ability to discuss and participate in palliative care [2]. Palliative care aims to improve quality of life and relieve suffering of patients facing life-threatening illness, as well as their family members, through a ‘whole-person’ approach to care that provides early identification, assessment and treatment of pain, as well as addressing all other physical, psychosocial, and spiritual issues [1]. Often used interchangeably, palliative care refers to care provided to all with life-limiting illnesses, while EOL refers to care provided to those who are terminal and predicted to die within the near future [1].

Due to demographic trends, access to palliative care is of growing interest among researchers and policy makers [5, 6]. Yet, much of the research examines access based upon a homogenously conceived normative population. Society, however, is comprised of a diverse array of individuals who are situated within social (e.g., gendered, racialized), economic, political, and cultural power hierarchies, which produce patterns of privilege and oppression for differing people and groups [7–9]. Through various relationships and effects of power, a person or population’s lower position within these social hierarchies can produce what has been referred to as ‘structural vulnerability’ (e.g., poverty, homelessness, etc.) [10], which, as a result, constrain choices and opportunities while amplifying vulnerability to risk, harm, and negative health outcomes [10, 11]. Through the lens of intersectionality [12–15], structurally vulnerable populations are defined in this study as people living in poverty and who are experiencing some level of homelessness, while at the same time are also experiencing various forms of racism, a history of or ongoing trauma and violence, social isolation, stigma associated with mental health issues, cognitive impairments, behavioural issues, substance use (previous or ongoing), interactions with the criminal justice system, and mobility issues and/or disability. Importantly, a population’s or person’s positionality as structurally vulnerable is not static, but dynamic in that it can change over time in response to external forces (e.g., policy reforms, economic restructuring) that affect their access to social and material resources [11].

While barriers to palliative care exist for normative populations, barriers experienced by those who are structurally vulnerable are likely amplified. Yet, little research has examined experiences of accessing health care services at the EOL by society’s most vulnerable groups: those with life-limiting illnesses *and* who are experiencing structural vulnerabilities [16–18]. This analysis draws on 30 months of ethnographic data collected to provide a contextual description of structurally vulnerable peoples’ experiences accessing health care services at EOL. The aim is to identify potential gaps in service provision and promote equitable provision of palliative care for *all* population groups.

### Death, dying, and access to care for structurally vulnerable populations

Impacted by forces of oppression, such as racism, colonialism, sexism, and/or classism, structurally vulnerable populations experience disproportionate rates of negative health outcomes, including, a heightened risk of poorer mental health, problematic substance use, as well as premature chronic morbidity and earlier death expectancy when compared to the average population [19–24]. Such negative health outcomes are the manifestation of various structural and systemic processes that produce violence, trauma, and harm [25, 26]. These processes constrain peoples’ agency and opportunities to achieve and maintain adequate health and access needed health care [27]. Although the level of systemic suffering and negative health and life expectancy outcomes of structurally vulnerable populations denote an increased need for care, barriers experienced in accessing such care are likely the highest [25], particularly at EOL. Existing disparities in access to palliative care emerge partly from deep-rooted assumptions regarding who needs such services [28]. The majority of those accessing palliative care tend to share similar socio-demographic and economic profiles (e.g., are diagnosed with diseases such as cancer, with more predictable trajectories, come from dominant social groups, have strong family and community connections, and are stably housed [16, 29, 30]. However, many dying people fall outside this demographic – those who are experiencing homelessness or unstable housing, poverty, mental illness and substance use, and stigmatized diseases such as HIV/AIDS and Hepatitis C. Their experiences remain largely unnoticed and their needs neglected [28].

Research on access to palliative care for structurally vulnerable populations [16, 31–34] suggests those who are homeless are unable to access palliative care until very late in their illness, if at all [35, 36]; they have relatively fewer social supports and often die alone in acute care, shelters, and transitional housing or less than ideal places such as alleys, streets, and vehicles [37–40]. Other studies indicate homeless people fear dying anonymously or undiscovered, are ambivalent about seeking out support, and significant challenges exist for discussing palliative care in these populations [16–18]. It was found by Ko et al. [41] that a fear of discrimination and negative emotions associated with EOL planning, stemming from previous life experiences and trauma, resulted in homeless older adults choosing not to seek medical care at the EOL. This body of research illuminates experiences that structurally vulnerable populations face in accessing care at EOL. However, deeper understandings of the complexities that ultimately shape access to care based on the experiences and perspectives of this population group are few.

## Methods

Ethnography informed by the critical theoretical perspectives of equity and social justice was employed. Ethnographic methodologies focus on qualitatively exploring the nature of particular socio-cultural phenomena in the environments where they occur [42]. Equity-based approaches aim to critically examine whether the distribution of resources, or outcomes of various social processes, are fair among diverse groups of people [27, 28]. Health inequities refer to those unfair, unjust, or potentially remedial differences in health or access to healthcare that result from structural arrangements [43]. Theories of social justice challenge inequities by critically examining complex social and power relations and the ways in which they contribute to the development of structural inequities [26–28]. From this theoretical positioning, the lens of intersectionality was also applied, which aims to explore the complex simultaneous and interdependent interactions between types of social difference and identity (e.g., gender, sex, race, socio-economic status, mental health, etc.), and forms of systemic oppression (e.g., sexism, racism, ableism, etc.) at micro and macro scales [9]. Ethnographers working from such critical perspectives are concerned not only with how social structures, processes, and ideologies work to constrain the lives of people, but also seek to generate knowledge that leads to social change [44]. Such an approach allows for the interpretation of individual healthcare experiences, while simultaneously considering the broad social, political, cultural, economic and historical relations that shape these experiences.

### Data collection

The study took place in a western Canadian province, with research ethics approval granted from university and health authority ethics committees. Participants consisted of three groups: (1) those experiencing structurally vulnerability and who were deemed to be on a palliative trajectory; (2) their support persons (e.g., ‘street family’); and (3) their formal service providers (e.g., housing workers, clinicians). Participants had diverse social locations with histories and ongoing experiences of racialization, colonization,^1^ trauma and violence which may have occurred in institutions (e.g., prisons, hospitals) and the ‘home’ (e.g., family home, foster care); stigma associated with mental health issues (not necessarily diagnosed); stigma and criminalization of past or current substance use and criminal justice involvement. Many participants also experienced mobility issues, cognitive impairments, learning disabilities, health literacy challenges, behavioural issues; and were a younger aging population (40–50 years).

Recruitment for the study was done in collaboration with two community-based organizations who work closely with structurally vulnerable populations. The recruitment process began with inviting health, housing, and social care service providers to participate via pamphlets, posters, and presentations at their places of employment. After obtaining consent, service providers were asked to assist with recruitment of palliative participants experiencing structural vulnerability by sharing information about the study and providing them with letters of invitation. Initially, services providers used the “surprise question” to assist with recruitment (i.e., would you be surprised if this person died in the next year?), which is commonly used to identify people with life-limiting conditions who could benefit from access to palliative care services [45]. However, this approach was aborted as many service providers did not have access to their clients’ medical charts or the medical knowledge to make such determinations and many expressed that they would not be surprised if *all* of their clients died in the next year. Recruitment then shifted towards referrals from clinicians (mostly physicians and nurses) working within community-based healthcare clinics and organizations. Diverse characteristics among participants experiencing structural vulnerability (e.g., illness/disease, gender, age, ethnicity, housing status, use of substance(s), mental health) were sought in order to capture maximum variation [46]. Support persons (e.g., family, friends, street family), when present, were invited to participate.

Participants included 25 people experiencing structural vulnerability, 25 support persons, and 69 formal service providers. Table 1 and Table 2 summarize characteristics of participants experiencing structural vulnerability and their support persons. All from this participant group were characterized homeless or vulnerably housed, under a Canadian definition associating level of income with housing stability [47]. As a result, they faced high rates of displacement (e.g., eviction, hospitalizations, moves) during data collection. Service provider participants’ varied in disciplinary background and length of time working in their current role with structurally vulnerable populations (see Table 3).

**Table 1:** Structurally vulnerable participants’ characteristics (*n* = 25)

Characteristic	Number of Participants
Gender	
Men	16
Women	9
Age ^a^	
Average age	59
Age range	19–81
Ethnicity ^b^	
White	13
Indigenous	8
African Canadian	1
Sexual orientation ^b^	
Heterosexual	20
Gay/Lesbian	2
Relationship status ^b^	
Single	8
Married or living in common law relationship	3
Divorced or separated	9
Widowed	2
Highest level of education ^a^	
University	2
College diploma	1
Some college (including trade school)	4
High school	6
Some high school	4
Middle school (grade 8)	2
Elementary school or less	2
Housing type on entry of the study	
Social or public housing	11
Market rental (with roommates and/or financial supplements)	8
Transitional housing (incl. Hotel/motel)	2
Homeless (e.g., shelter, boat, hospital, etc.)	4
Main source of income^a^	
Provincial Disability Benefit	13
Pension	6
Social assistance	1
Employment Insurance Benefit	1
Primary life-limiting condition of concern	
Cancer	15
Chronic Obstructive Pulmonary Disease (COPD)	2
Diabetes	3
Unknown ^c^	5
*Other health conditions*	
Arthritis	10
Cardiovascular disease	6
HIV/AIDS	2
Hepatitis C	6
Mental health status	
Self-reported mental illness	7
Mental illness identified by health service provider (including undiagnosed but suspected)	7
Access to health care	
Had regular medical doctor	23
*Care sites most commonly accessed*	
Primary care clinic	18
Other medical clinic/hospital	7
*Access to palliative care* ^d^	
Over 2 weeks of palliative care	5
2 weeks and under of palliative care	5
No access to palliative care	3
Deaths	
Number of deaths during study period	13
*Place of death*	
Home	8
--supported housing	6
--market housing	2
In-patient palliative care unit	5
*Accompaniment at death*	
Alone	5
With family member	6
With service provider	2

^a^Based on 21 participants. Three structurally vulnerable participants engaged in observations but died prior to completing a demographic form. One participant did not answer this question

^b^Based on 22 participants. Three structurally vulnerable participants engaged in observations but died prior to completing a demographic form

^c^Five participants were not identified (either self or by service providers) as living with a life-limiting condition

**Table 2:** Supporter participants’ characteristics (*n* = 25)

Characteristic	Number of Participants
Gender	
Men	11
Women	14
Age^a^	
Average age	50
Age range	35–71
Ethnicity^a^	
White	12
Indigenous	6
Sexual orientation^a^	
Heterosexual	16
Gay/Lesbian	2
Relationship status^a^	
Single	9
Married or living in common law relationship	6
Divorced or separated	3
Widowed	0
Relationship to structural vulnerable person	
Friend/Street family	10
Biological family	10
Current or former partner	5
Highest level of education^b^	
Post graduate degree	2
University	1
Attended university	1
College diploma	1
Some college (including trade school)	5
High school	2
Some high school	4
Middle school (grade 8)	1
Elementary school or less	0
Housing type on entry of the study ^a^	
Purchased home	2
Social or public housing	2
Market rental (with roommates and/or financial supplements)	11
Homeless (e.g., shelter, boat, hospital, etc.)	3
Main source of income^a^	
Provincial Disability Benefit	3
Pension	4
Social assistance	4
Employment Income	6
Employment Insurance Benefit	0
Other	1
Life limiting conditions^b^	
Cancer	1
COPD	3
Diabetes	1
Cardiovascular Disease	3
Arthritis	5
*Other conditions* ^b^	
HIV/AIDS	1
Hepatitis C	4
*Mental health status* ^b^	
Self-reported mental illness	5

^a^Based on 18 participants. Seven supporter participants engaged in observations but were lost to follow up and did not complete a demographic form

^b^Based on 17 participants. Seven supporter participants engaged in observations but were lost to follow up and did not complete a demographic form. One participant did not answer this question

**Table 3:** Service provider participants’ characteristics (*n* = 69)

Characteristics	Number of Participants
Gender	
Men	25
Women	41
Other	3 ^a^
Age^b^	
Average age	44
Age range	24–67
Highest level of education^c^	
Post graduate degree	6
University degree	23
Attended university	5
College diploma	5
Some college (including trade school)	4
High school	0
Some high school	1
Middle school (grade 8)	0
Elementary school or less	0
Employment role	
Outreach/support worker	16
Physician	15
Nurse	13
Housing worker	7
Counsellor/social worker	5
Manager/coordinator	4
Other	9
Length of time in current employment role^d^	
Less than 1 year	2
1 year to 5 years	20
6 years to 10 years	6
11 to 15 years	5
16 years to 20 years	2
20 +	5

^a^Other responses are: Genderqueer, Two-Spirit, Trans

^b^Based on 43 participants. Twenty-five engaged in observations but were lost to follow up and did not complete a demographic form. One participant did not answer this question

^c^Based on 44 participants. Twenty-five engaged in observations but were lost to follow up and did not complete a demographic form

^d^Based on 40 participants. Twenty-five engaged in observations but were lost to follow up and did not complete a demographic form. 4 participants did not answer this question

Data collection with structurally vulnerable participants, their support persons and service providers included repeated participant observation over a 30-month timespan; 300 h of observation were conducted in homes, shelters, transitional housing units, community-based service centres, on the street, and at health care appointments. The relationship between the researchers collecting data and participants was not pre-existing; yet, all researchers conducting data collection held experience working with structurally vulnerable populations. Structurally vulnerable participants were provided a cash honorarium of $20 CAD cash or the financial equivalent in food and/or nutritional supplements for each observation session.

Observations were conducted by research staff, around-the-clock and throughout the week, without restrictions. Documenting access to healthcare services for those experiencing structural vulnerability on a palliative trajectory was the goal of observational data collection, as well as gaining a better understanding regarding the context from which participants made decisions about if, when, how, and where to seek their care. Observational field notes aimed to capture the setting, people, activities, signs (clues that provide evidence about meaning and behaviour), acts (what people are doing), events, time, goals (what people are trying to accomplish), and connections of structurally vulnerable participants on a palliative trajectory. Field notes also included reflexive notes of researchers, such as their thoughts about the observation, potential influences shaping the observation, and any notes for future observations/interviews. Field notes were recorded by hand and then transcribed. In-depth interviews were conducted to supplement observational data, which allowed for clarification and validation on what was being observed. The questions used in these interviews were context specific and included asking participants, for example, to describe a health care interaction that was just observed and how they felt about it or how they came to making their decision regarding how, where, and when they chose to access particular care. Interviews (*n* = 19 structurally vulnerable participants; *n =* 16 support persons; and *n* = 23 service providers were recorded digitally, transcribed verbatim. These interview transcripts, along with all observational fieldnotes, were then entered into NVivo™ for analysis.

### Analytic technique

Analysis of the data was conducted by the entire research team who met repeatedly throughout the data collection process to identify emerging themes and issues to consider for further investigation. The constant comparative method was used, which involves data collection and analysis occurring concurrently [48]. Using a social justice and equity lens, broad themes were identified by the research team, and upon reaching consensus, coding of the data began. Coding of the data was conducted by a team of three researchers, two of whom were also involved with data collection. The coding process involved first coding and then recoding data using an inductive process of organization, where incidents or themes are compared to other incidents or themes. To do this, we began with open coding to generate broad categories (e.g., barriers to care; facilitators to care) that were then refined and recoded [48, 49].

This analysis is based upon data coded into the overarching theme of ‘barriers to care’. Using this data, a more refined thematic analysis unveiled that barriers to care were due to structural/social (e.g., poverty, criminalization), organizational/institutional (e.g., continuity of care, professional communication) and individual level (e.g., avoidance, behaviours) issues.. Again, the research team met regularly during this analytic process to review data and emerging themes, which enhanced analytic rigor via investigator triangulation. To ensure anonymity, participant pseudonyms have been used.

## Results

Five themes regarding barriers to accessing care emerged: (1) survival imperative; (2) normalization of dying; (3) the problem of identification; (4) professional risk and safety management; and (5) cracks of a ‘silo-ed’ care system. Although presented as five separate themes, these barriers were interconnected, and the result of various forces ranging in scale across structural/systemic, organizational/institutional, and individual levels.

### The survival imperative

Participants experienced social disadvantages and oppressions (e.g., poverty, inadequate housing, racism) which limited their capacity to access care. When access to immediate needs for shelter and food were lacking, accessing palliative care services was not the priority: rather, daily survival was. Acquiring enough food was an everyday challenge for Amber. As reflected in fieldnotes: “*It became clear that her [Amber’s] focus all day, every day, was simply on finding food*”. Accordingly, participants viewed many aspects of palliative care, like attending medical appointments, as secondary to the demands associated with meeting more immediate needs. Rachael, a physician, described that a major barrier occurred because people experiencing structural vulnerabilities are just so *“busy living in the moment and surviving”.* Thus, advance care planning, provision of palliative care, or discussions about death and dying were simply absent from participants’ everyday lives; awareness of and knowledge about palliative care services potentially available to them was either minimal or non-existent. This lack of awareness was similarly reflected by many community-based service providers who also had little to no knowledge of what palliative care was or what it could offer in support to their clients.

Some participants sought care from the formal healthcare system to meet their palliative needs, but, with few exceptions, such care did not acknowledge the burden placed on them by pressures to meet basic survival needs. This amplified participants’ vulnerability as they were unable to physically seek out their daily needs for survival as they became increasingly ill. George was socially isolated, and with limited material resources, could not afford transportation to medical appointments associated with his end-stage liver disease. As he became increasingly frail and in pain he required medical care but when he discussed his financial concerns with his physician his needs went unacknowledged even though this was the major barrier that George faced in accessing care. Observations revealed that health care providers rarely acknowledged the everyday requirements that people need to survive (i.e., food, shelter, income) and how this might influence their ability to access services and have their palliative care needs met. In the context of a biomedically-focussed health care system, it was observed that health care providers appeared to perceive these more social care needs as falling outside their scope of practice. Addressing deficits in housing, food security, income, transportation and other social dimensions of health was an essential component of palliative care for participants and it was only when these issues were addressed that participants were more likely to obtain quality care at EOL.

### The normalization of dying

Participants had long histories of surviving social disadvantages, such as poverty, homelessness, colonization, stigma and discrimination, social exclusion and marginalization. They had witnessed many people in their social networks dying and many had repeatedly been told they were going to die as a result of their ‘lifestyle’. This discourse caused a certain amount of ‘normalization’ of death in the community. As one outreach worker said: “*Everybody in this community is at risk of dying.”* This sentiment was likely perpetuated by the unprecedented number of drug overdose deaths that were occurring across the province at the time of the study. Death by drug overdose in the street community had become a daily occurrence. When participants were told they were on a palliative trajectory, they often did not react with the kind of concern that is the expected norm. Sherry had been told multiple times that she was at risk of dying due to her addictions. Once diagnosed with metastatic cancer, she did not take her diagnosis, the medical system, or the care she required seriously. It was not until she was in severe pain from her cancer that Sherry was convinced by her case manager to seek help. One participant who managed a transitional housing complex explained that death is expected in the street community and that it does not come as a surprise:

We assume that most of the people we work with in housing are close to end of life. That’s their health, coupled with their lifestyle or behaviours around drug and alcohol use. That combination [means] they’re probably quite close to risk around end-of-life. So, it’s never a shock.

This normalization of death in the street community as a result of the high number of drug overdose deaths combined with an assumption that lifestyle factors would result in death contributed to the challenge of identifying those people who were structurally vulnerable and also had ongoing palliative needs. Palliative care needs were relatively invisible as a result.

### The problem of identification

Relatedly, identification of those who were dying and could benefit from palliative care was challenging in a system where much of the care was provided by workers outside the formal medical system (i.e., social service, outreach and housing workers). These workers had limited health or palliative care knowledge, experience, or training. Unless participants were being actively case-managed by health care providers, they were generally not identified. This was evident when attempting to recruit participants. In almost all cases, study referrals from community workers resulted in participants who were chronically ill with multiple co-morbidities, but on further assessment by our team, were not people who would be expected to die in the next year. Even when participants were accessing health care services, they were not guaranteed to be identified as in need of a palliative approach. Observations exposed instances of symptoms going unmanaged and sometimes of people dying in a less than humane manner. Sammy died alone, lying in his own vomit, on the floor of his single room occupancy residence. Clear symptoms and signals regarding the palliative care needs of those experiencing structural vulnerability were found to often remain unnoticed. This was found to often be due to the challenging and complex context from which social service providers (and in some instances, health care providers) are to identify, within a meaningful timeframe, those with life-limiting diagnoses and on a traditionally defined ‘palliative trajectory’.

In contrast, when participants were identified and connected to providers with a palliative orientation and who understood the importance of addressing social determinants of health, access to palliative care improved; services tended to come around them quickly and efficiently. For Cliff, being identified resulted in “*top rate service”:*

I am surprised at how much is actually available to me, and how well I’ve been treated. And since I got the cancer, it’s been nothing but positive reaction from anything I do need or wherever I’ve had to go to get help. They’ve [service providers] been more than accommodating. Like, I mean, I’m getting top rate service. They fast tracked me through the system for any [palliative] benefits … It’s just anything I need is actually there for me. And it’s been made quite clear, just call if you need anything. Yeah, I feel like I’ve been taken care of very well at this point.

By virtue of being identified as in need of palliative care, these participants experienced care in ways they never had, including feeling believed, getting their pain needs met, having access to additional income and services, and being surrounded by care providers who were compassionate, kind and invested in their care. However, it was not until (or if) they were identified as dying that access to such care was ever received, and for those who were identified, they were mostly diagnosed with cancer, the more typical palliative care diagnosis.

### Professional risk and safety management

Contemporary Canadian palliative care policy is largely directed towards increasing supports for ‘home deaths’ [5, 50, 51]. The outcome of this policy directive, however, is that those who are dying and experiencing structural vulnerability, particularly homelessness, have increasingly limited options in where they can access palliative care. It was found that even for those who are ‘housed’, access to community/home-based care was found to be denied based upon assumptions of what a safe and secure home is or should be.^2^ The intersection of structural and individual vulnerabilities created a context whereby participants’ lived realities were often perceived by institutions and/or service providers as unsafe or risky. Community-based service providers also shared that their clients had health services discontinued even when housed because of safety policies that prevented care from being delivered in settings deemed risky (e.g., overcrowded, where cigarette smoke,^3^ drugs, or drug-use equipment was present, or where violent incidents had occurred). Jonathan, a housing worker, implied that such risk management policies stem from and reinforce structural stigma that constrains access to care even when actual risks were negligible:

A lot of folks that we house, drink and use whatever form of substances. There’s a lot of kickback from [home support services]. “I’m not going to go into a place where there’s smoke. I’m not going to go into a place if there’s an open bottle of alcohol. I’m not going to go in.” There’s a lot of judgement and stigma. Even though there is absolutely no safety concerns, there is judgement that is being passed that says, “I do not work with someone like that.”

Stigmatization of substance use and mental health issues were found to influence access to care. A lack of affordable and adequate housing, combined with risk management policies, meant that people could not ‘age-in-place’ and were moved (most often into acute care) as their care needs increased or as they approached EOL. Many participants reported they preferred to stay in their community as they approached EOL, surrounded by familiar providers and support people they trusted. However, being housed in spaces deemed ‘unsafe’ (i.e., single room occupancy hotels, supportive housing, or shelters) meant they had limited or no access to home health services that would enable their palliative needs (such as pain management) or other health needs to be met. Exacerbating vulnerability, in some cases, care was completely restricted except where individual homecare providers went against institutional policies to provide care ‘under the grid.’ In many instances, it was observed that these workers went ‘above and beyond’ their job descriptions in effort to keep participants in the community, including doing personal care and providing medications. Going against such policies put providers at risk themselves because they were doing work outside of their scope of practice or their institutional rules; many experienced distress and frustration as they bore witness to gaps in care, inequities, and injustices:

Yeah, anger and frustration and disappointment and resentment, all those kinds of negative feelings. And then, a little bit of guilt. Like here is somebody [who is dying] who’s somebody’s mother, daughter, sister, your client. You’ve known them forever and they’re getting substandard care and you feel really shitty that you can’t seem to make a difference. Yeah, you can’t seem to change the system.

Risk management policies, while put in place with good and reasonable intentions, resulted in major barriers to accessing needed palliative care. Combined with decreasing mobility and increasing symptomatology at EOL, these policies served to amplify structural vulnerabilities, social isolation, and marginalization.

### The cracks of ‘silo-ed’ care systems

Health and social service systems in Canada operate within defined boundaries regarding roles and responsibilities, which results in ‘cracks’ between these systems through which those who are structurally vulnerable fell. Although widely recognized, such cracks created tensions and uncertainty about who was responsible for meeting unmet palliative care needs. While barriers to accessing palliative care were frequently identified in the data, the question of whose responsibility it was to galvanize an initiative that would address access challenges was a critical barrier. Those working within social services did not always view the delivery of palliative care within their mandate, nor did they feel qualified to provide such care. Dennis, a housing worker, explained: *“This is a supported housing building, so we are limited around what we can provide around medical support. We don’t have nursing staff, we don’t have medical alerts, we’re not set up that way.”* Other social service providers acknowledged that they were already providing such care indirectly and informally and were going beyond professional roles to ‘fill’ existing cracks and provide required support.

The silo-ing influence of health and social services also means participants had to navigate through multiple departments, organizations, and agencies, challenging as they became increasingly frail, in pain, and fatigued. Our participants required access to a greater number of supports, both social and health related, than the general population while at the same time generally held lower levels of health literacy, educational attainment, cognitive capacity as well as limited access to material resources (e.g., phones, internet), transportation, care coordination, and informal caregiving support made navigating the maze of health and social care a disproportionately arduous task. With little informal support, participants were often left to navigate and coordinate their care alone. Even when they had a support person in their lives, it was observed that navigation was a major challenge. Both Dani and her wife Sharron had been diagnosed with a life-limiting condition and found it difficult to keep track of appointments in the context of structural vulnerability and functional decline. Their challenges were documented in an observational fieldnote:

They were told many different things and there were many different places they were supposed to go, and they couldn’t make sense of it. It would have been helpful, they told me, to have someone to keep track of it for them, and to tell them where they needed to be and when—possibly even to help them get there.

Due to the complexity of the care system, many participants failed to receive the palliative care and support they needed as they did not know who to see, where to go, and when to go to find care.

With silo-ed care systems came a lack in continuity of care providers. Issues with continuity of care were especially problematic for those who had experienced stigmatization, colonization, racialization and other forms of injustice. Felix explained how he had built strong social defenses to protect himself from harm and found it challenging to trust others. Once diagnosed with cancer and needing increased levels of care, continuity of care across providers was critical but not achieved. With continuity of care lacking, the development of trusting relationships, an essential component of quality palliative care, did not occur. Likewise, George explained that discontinuity made it difficult to keep track of his care providers and what he was told: “*I don’t remember all these people. I’ve got so many social services coming at me and doctors and nurses and I can’t remember all their names and all their details [of information]”*. If participants did access care systems, gaps in care due to a lack of provider continuity became apparent, as participants received ‘fractured’ care, often with little guidance and/or meaningful explanation regarding why they were dying, what to expect, or why they needed particular care, tests, or procedures done.

## Discussion

Although this analysis focusses on barriers to accessing care at EOL, and while the complexities were many, our participants were not passive victims of vulnerability, but were also highly resilient. Many had developed expert survival skills, were extremely resourceful in finding ways to manage their pain in environments of stigma, discrimination and criminalization, and some were able to create supportive networks. In this sense, some had found ways to attend to EOL care needs themselves. Despite such strength and resilience, the common experience among participants was characterized by a disproportionate level of barriers to accessing care. These barriers were not experienced in static ways, but rather exacerbated their vulnerability as they moved across the life course and approached EOL.

Taken together, findings demonstrate that structurally vulnerable populations face significant barriers in having their palliative needs met, including a broader focus on the social determinants of health. One’s positionality within the broader social hierarchies of power not only shapes one’s ability to obtain and maintain health, but also access to quality care at EOL. Due to this lived context, service providers faced significant challenges in identifying those who were dying and in need of palliative care resulting in participants either receiving care too late, or not at all. This is consistent with a recent study by Shulman et al. [34] pointing toward the complexities of identifying who is in need of palliative care. For participants in this study, including care workers and those who were identified as needing palliative care, however, they were found to be desensitized to death, dying, and prognosis, which constrained their capacity to follow up with needed care. Our findings also suggest that when structurally vulnerable people are identified and attempt to access care services, they will often face additional barriers as a result of risk management policies and silo-ed health and social care systems that result in discontinuity of care and system navigation issues.

Overall, findings from this analysis unveil inequities that exist in accessing and receiving care at EOL, despite equity in access being a mandate of most westernized health care systems [52] and that access to palliative care is increasingly recognized as a human right [53, 54]. Those who do not fit into the normative palliative patient population for whom palliative care programs and policies are currently built will face significant barriers in accessing quality EOL care [50, 55–58]. As palliative care has been largely designed to service the normative palliative population with cancer diagnoses and relatively predictable trajectories, the needs of those with differing diagnoses (e.g., non-cancer diagnosis) and lived contexts, including structural, social, organizational, and individual conditions, are not being addressed. These access barriers highlight the shortcomings of a ‘one-size fits all’ or ‘blanket’ policies that fail to recognize diversity, such as lack of access to basic daily requirements for survival, and not considering *who* needs *what kinds* of support. For example, many palliative programs and policies promote enabling care to take place in the home; however, this policy directive has multiple assumptions, including that everyone has a safe and secure home, access to the required associated material resources, and access to friends or family who are both able and willing to provide care at home. While many people, including the structurally vulnerable, may wish to spend the end of their life at home with family around them, it is necessary to unpack what is being defined as home and family in such policy discourse to identify who is left out. Considering this, it becomes apparent that dominant approaches to palliative care policy are not adequately acknowledging diversity, and particularly, population groups who are structurally vulnerable. As such, there is a need for more palliative care research to examine and articulate structural vulnerabilities to consider and address existing inequities and underlying power structures that reinforce them. Questions also remain related to whether certain structurally vulnerable groups (e.g., those with cancer) have better access to palliative care than others (e.g.,, non-cancer diagnosis).

How do we address palliative care needs in a population that is living in the moment to survive, who is desensitized to death, socially excluded, and facing a system that poses major navigational problems? Current palliative care models must be re-envisioned to reach out and meet people where they reside. At the systems level, greater support for developing partnerships between medically-based palliative care professionals and community-based social service providers would serve to enhance access to and reduce barriers to quality palliative care. At the meso- level, there is a need for organizations and departments to become more attuned to the significance of continuity of care, including consistency in the assignment of care providers. At the micro-level, there is the need to support social service providers to expand their education and training to become better equipped to identify those in need of palliative care. Concurrently, health care providers require enhanced capacity to acknowledge and consider the impacts of the social determinants of health in their provision of EOL care, as well as the importance of developing trust/respect/dignity when working with particular population groups. While a paucity of research exists on this issue, some notable efforts are currently underway [35]. Within Canada and the United Kingdom, initiatives such as the Palliative Education and Care for the Homeless (PEACH) program [59], the Calgary Allied Mobile Palliative Program [60] and the Palliative Care Service at St. Mungo’s [61] aim to provide quality palliative care for the homeless and vulnerably housed, based on principles of equity, justice and harm reduction. Shelter-based palliative care services have also been developed [62], though these are not widespread. These are positive steps forward toward developing strategies that employ an inter-sectoral approach to enable community-based agencies and formal palliative care providers to work together to shape action and interventions.

Like everyone, people who are structurally vulnerable deserve the highest quality care at the EOL. However, they do not always receive it and sometimes die in conditions that are less than ideal. Our findings underscore that without recognizing structural vulnerability, the advancement of equitable access to quality EOL care remains, in large part, an unachievable goal. Greater attention to such lived realities is needed, while also recognizing how structural vulnerability, and resulting marginalization, becomes amplified as one moves across the life course [11]. As such, our findings highlight the need for initiatives that are flexible, inclusive, accommodating, and cater to the needs of those who are unstably housed, living in poverty, socially marginalized, and have or continue to experience various forms of structural and individual level violence.

## Conclusion

Care of the dying and ensuring that their needs are met is inherently complicated. In the presence of conditions such as homelessness, severe mental illness, high incidences of substance use and histories of trauma, new and innovative approaches to care are required that take into account the social determinants of health and the factors that influence access to care. Such approaches will help to minimize social-structural inequities, and address the unique palliative care needs of structurally vulnerable populations.

## Abbreviation

EOL: End-of-life

## Footnotes

1 In the Canadian context, colonization refers to the policies and practices of removing Indigenous peoples’ from their land, culture and community. For instance, residential schools for Indigenous people in Canada date back to 1870 with the last school closing in 1996. Over 130 residential schools were located across the country, which were government-funded and church-run, and set up to eliminate parental involvement in the intellectual, cultural, and spiritual development of Indigenous children [63].

2 All participants were characterized as homeless, which by the Canadian Observatory on Homelessness [64]. include a range of housing and shelter circumstances. For example, some participants were unsheltered (e.g., absolutely homeless or living on the street); Emergency sheltered (e.g., staying overnight in shelters for those who are homeless or impacted by family violence); provisionally accommodated (referring to those whose accommodation is temporary or lacks secure tenure); or at risk of homelessness (e.g., those whose economic/housing situation is precarious. In our study, some participants resided in supportive housing, which in British Columbia, involves a form of provisional accommodation by a non-profit housing provider, which offers a range of on-site, non-clinical supports such as life-skills training, connections to primary health care, and mental health or substance use services [65].

3 Within the British Columbian city where the study took place, policies exist which aim to protect community-based health support providers from risks, including second-hand smoke. In this jurisdiction, service provision is limited to two hours before or two hours after a person has had a cigarette in their home. This was found to create a number of challenges as many participants were active smokers and often did not remember the dates and times providers were scheduled to arrive. Furthermore, specific times were often not given by providers as their schedules often shifted in response to their clients and the care required of them on those days.
